# SUMOylation of ZEB1 Modulates PANoptosis in Burn‐Induced Early Acute Kidney Injury

**DOI:** 10.1111/jcmm.70865

**Published:** 2025-10-22

**Authors:** Juncheng Zhang, Zhengjie Huang, Qin Lin, Hongbin Zhong, Weiping Hu, Fengling Zhang, Jiyi Huang

**Affiliations:** ^1^ The School of Clinical Medicine Fujian Medical University Fuzhou Fujian China; ^2^ Department of Gastrointestinal Surgery, The First Affiliated Hospital of Xiamen University, School of Medicine Xiamen University Xiamen China; ^3^ Department of Nephrology, Xiamen Key Laboratory of Precision Diagnosis and Treatment of Chronic Kidney Disease The Fifth Hospital of Xiamen Xiamen Fujian China; ^4^ Department of Nephrology, Fujian Clinical Research Center for Chronic Glomerular Disease, The First Affiliated Hospital of Xiamen University, School of Medicine Xiamen University Xiamen China

**Keywords:** acute kidney injury, cell death, hypoxia/reoxygenation, SUMOylation, ZEB1

## Abstract

Burn‐induced acute kidney injury (AKI) involves inflammatory programmed cell death pathways, including PANoptosis. This study investigated how SUMOylation of the transcription factor ZEB1 regulates PANoptosis in early burn‐induced AKI. Human AKI datasets were analyzed for ZEB1 expression and correlation with PANoptosis/SUMOylation genes. A rat burn‐AKI model (24–72 h) evaluated renal dysfunction (creatinine, urea), histopathology (H&E, TUNEL) and oxidative stress (MDA/SOD/CAT). In vitro, HK‐2 cells under hypoxia/reoxygenation (H/R) assessed ZEB1 and PANoptosis effectors (Caspase‐3/MLKL/GSDMD). Co‐immunoprecipitation assessed ZEB1 SUMOylation and ubiquitination. Flow cytometry measured intracellular reactive oxygen species (ROS) levels. ELISA quantified IL‐1β and IL‐6. Functional studies used ZEB1 knockdown, SUMOylation inhibition (2‐D08) and SENP1 overexpression. Bioinformatic analysis revealed ZEB1 upregulation in human AKI correlating with PANoptosis/SUMOylation genes. Burn‐AKI rats showed significant renal dysfunction, injury, oxidative stress (peak 48 h), increased ZEB1/PANoptosis effectors and enhanced ZEB1 SUMOylation (reduced ubiquitination). Similar findings were observed in H/R‐treated HK‐2 cells. SENP1 overexpression reversed these changes. ZEB1 knockdown or SUMOylation inhibition improved cell viability and suppressed oxidative stress, inflammation and PANoptosis effectors. H/R‐induced ZEB1 SUMOylation is reversed by SENP1 or 2‐D08. ZEB1 SUMOylation promotes PANoptosis in burn‐induced AKI, representing a potential therapeutic target for early intervention.

## 1 Introduction

Acute kidney injury (AKI), characterised by a rapid decline in renal function, is a common and life‐threatening complication in burn patients, affecting 9%–50% of individuals within 72 h post‐injury and significantly increasing the mortality risk [1, 2]. Burn‐induced AKI arises from systemic hypoperfusion, oxidative stress and inflammatory cascades, which collectively drive renal tubular epithelial cell injury, a central event in AKI pathogenesis [3]. While these upstream insults are recognised, the molecular links to tubular cell death and dysfunction are not fully understood, hindering targeted therapy development.

Emerging evidence implicates PANoptosis, a coordinated, inflammatory form of programmed cell death (PCD) that integrates pyroptosis, apoptosis and necroptosis, as a critical driver of tubular injury in kidney diseases [4, 5]. Unlike isolated PCD pathways, PANoptosis is orchestrated by supramolecular complexes, such as PANoptosomes, which coordinate caspase and receptor‐interacting protein kinase activation to promote inflammatory tissue injury and fibrosis [6, 7]. In burn‐induced AKI, reactive oxygen species (ROS) and proinflammatory mediators likely initiate PANoptosis, but the upstream regulatory mechanisms of this process remain poorly understood [7, 8].

Zinc finger E‐box‐binding homeobox 1 (ZEB1), a transcription factor implicated in epithelial‐mesenchymal transition (EMT) and renal fibrosis, has emerged as a potential modulator of tubular injury [9]. Prior studies have shown that ZEB1 knockdown attenuates fibroblast activation [10], reduces apoptosis [11] and preserves renal epithelial cell proliferation [12] in experimental AKI models. Notably, ZEB1 can transcriptionally activate gasdermin E (GSDME), a pyroptosis effector, during EMT, suggesting its involvement in inflammatory PCD [13]. Despite these findings, whether ZEB1 regulates the integrated PANoptotic response in burn‐induced AKI and how its activity is modulated remains unknown.

Post‐translational modifications such as SUMOylation, ubiquitination and phosphorylation play essential roles in regulating transcription factor function, particularly under stress conditions [14]. Among them, SUMOylation, a reversible covalent attachment of small ubiquitin‐like modifier (SUMO) proteins, has been increasingly recognised for its role in modulating transcriptional activity, subcellular localization and protein stability in stress‐related signalling pathways [15]. Recent studies have shown that SUMOylation can modulate the activity of EMT regulators and influence apoptosis and inflammatory responses [16]. Given the role of ZEB1 in stress adaptation and PCD, we postulated that its function may be modulated through SUMOylation during AKI.

To explore this, we first examined public transcriptomic datasets to assess the clinical relevance of ZEB1 and its potential association with PANoptosis‐ and SUMOylation‐related genes in human AKI. Given that early burn‐induced AKI is predominantly driven by ischemia–reperfusion (I/R) injury due to systemic hypoperfusion followed by fluid resuscitation [17, 18], the hypoxia/reoxygenation (H/R)‐treated HK‐2 cell model was employed as a well‐established in vitro analog of renal I/R injury [19, 20]. Using both this H/R model and a rat burn model, we investigated the role of ZEB1 SUMOylation in PANoptosis activation. This study aims to elucidate a novel regulatory mechanism in burn‐induced AKI and identify potential therapeutic targets.

## 2 Materials and Methods

### 2.1 Bioinformatic Analysis

Transcriptomic data from two publicly available datasets, GSE53769 (AKI: *n* = 8, control: *n* = 10) and GSE139061 (AKI: *n* = 39, control: *n* = 9), were downloaded from the Gene Expression Omnibus (GEO) database to evaluate ZEB1 expression in AKI. ZEB1 expression in normal human tissues, including the kidney, was assessed using data from the Genotype‐Tissue Expression (GTEx) project. Pearson correlation analysis was performed in the GSE139061 dataset to evaluate associations between ZEB1 and canonical PANoptosis‐related genes (MLKL, GSDMD, CASP3), as well as SUMOylation‐associated genes, including small ubiquitin‐like modifier 1 (SUMO1) and SUMO1/sentrin‐specific peptidase 1 (SENP1). Gene interaction networks were constructed using the GENEMANIA plugin in Cytoscape [21] to predict functional connectivity between ZEB1 and SUMOylation‐related targets.

### 2.2 Animals

Female Sprague–Dawley (SD) rats (8 weeks old, 220–250 g, specific pathogen‐free grade) were obtained from Beijing Huafukang Biotechnology Co. Ltd. (Licence No. SCXK (Beijing) 2024‐0001). Animals were housed in a controlled environment (temperature 20°C–26°C, relative humidity 40%–70%, 12‐h light/dark cycle) with ad libitum access to food and water. Rats were randomly assigned to four groups (*n* = 6 per group): a control group and three burn‐induced AKI model groups corresponding to 24, 48 and 72 h post‐injury. All animal procedures were approved by the Institutional Animal Care and Use Committee of Jiangxi Zhonghong Boyuan Biotechnology Co. Ltd. (Approval No. LL‐202407050001).

### 2.3 Establishment of Burn‐Induced AKI Model

Rats were anaesthetised via intraperitoneal injection of sodium pentobarbital (45 mg/kg). After dorsal hair removal, a burn injury was induced using a hot water immersion method. For model groups, the exposed dorsal skin was immersed in boiling water (100°C) for 15 s, whereas control animals were treated with water at 25°C for the same duration. Following injury, rats were returned to their cages for recovery under standard conditions. At the designated time points (24, 48 and 72 h post‐injury), animals were sacrificed under deep anaesthesia by exsanguination.

### 2.4 Sample Collection

At 24, 48 and 72 h post‐injury, blood and kidney tissue samples were collected. Blood samples were centrifuged at 3000× *g* for 10 min at 4°C to obtain serum. Kidney tissues were immediately frozen in liquid nitrogen and stored at −80°C for subsequent analyses.

### 2.5 Cell Culture and Treatment

To recapitulate the I/R injury component of burn‐induced AKI [17, 18], we used H/R‐treated HK‐2 cells, a widely accepted in vitro model for renal I/R injury [19, 20], to mimic the hypoxic and oxidative stress conditions experienced by tubular epithelial cells following burn injury. Human renal proximal tubule epithelial cells (HK‐2; #iCell‐h096; iCell, China) were cultured in DMEM/F12 medium (#KGL1201‐500; KeyGEN BioTECH, China) supplemented with 10% fetal bovine serum (FBS; #10099‐141; Gibco, USA) and 1% penicillin–streptomycin (#15140122; Gibco) at 37°C in a humidified atmosphere of 5% CO_2_. To determine optimal H/R conditions, HK‐2 cells were exposed to hypoxia (94% N_2_, 1% O_2_, 5% CO_2_) for 24 h using a specialised CO_2_ incubator (BPN‐80CW; Shanghai Yiheng Scientific Instruments, China), followed by reoxygenation under normoxic conditions for 0, 1, or 3 h. Cells were then harvested for further analysis.

### 2.6 Cell Transfection

Transfection was performed when HK‐2 cells reached approximately 70% confluence. The culture medium was replaced with 1 mL of serum‐free medium. Two sterile microcentrifuge tubes were prepared: one containing 125 μL Opti‐MEM (#31985070; Gibco) mixed with 5 μL Lipofectamine 3000 (#L3000015; Invitrogen, USA), and the other containing plasmid DNA diluted in 125 μL Opti‐MEM. After 5 min of incubation at room temperature, the contents of both tubes were combined, mixed thoroughly and incubated for an additional 15 min to form liposome‐DNA complexes. The transfection mixture was then added dropwise to cells in a 6‐well plate. After 4–6 h of incubation at 37°C in a 5% CO_2_ atmosphere, 1 mL of complete medium containing 20% FBS was added to each well. Cells were maintained under standard culture conditions, and assays were conducted 48 h post‐transfection.

### 2.7 CCK‐8 Assay

Cell proliferation was assessed using the Cell Counting Kit‐8 (CCK‐8; #KGA317, KeyGEN BioTECH, China). Cells were seeded into 96‐well plates at 48 h post‐transfection. At the indicated time points, the medium in each well was replaced with 100 μL of fresh culture medium, followed by the addition of 10 μL CCK‐8 reagent. Plates were incubated at 37°C for 2 h. Absorbance was measured at 450 nm using a microplate reader (#WD‐2120B; Beijing Liuyi Biotechnology, China). All experiments were performed in triplicate.

### 2.8 Flow Cytometry Analysis

Apoptosis was assessed using Annexin V‐FITC/PI double staining with the Annexin V‐FITC/PI Apoptosis Detection Kit (Cat# AP101‐100‐kit, Multi Sciences, China). Cells were harvested, washed twice with phosphate‐buffered saline (PBS) and centrifuged at 1500 rpm for 3 min. The pellet was resuspended in 500 μL of pre‐cooled binding buffer, followed by the addition of 5 μL of Annexin V‐FITC and 10 μL of propidium iodide. Samples were incubated at room temperature in the dark for 15 min, then analyzed by flow cytometer (NovoCyte 2060R; ACEA Biosciences, Hangzhou, China).

Intracellular ROS levels were measured using a ROS detection kit (#KGT010‐1; KeyGEN BioTECH, China) following the manufacturer's protocol. Briefly, cells were incubated with 10 μM DCFH‐DA, freshly diluted in serum‐free medium, at 37°C for 20 min. After incubation, cells were immediately analyzed by flow cytometry. All flow cytometry experiments were performed in triplicate.

### 2.9 Haematoxylin and Eosin (H&E) Staining

Kidney tissues from rats were fixed in formalin, dehydrated through a graded ethanol series, embedded in paraffin and sectioned at 4 μm thickness. Sections were stained with haematoxylin and eosin (#ZLI‐9610; Zhongshan Golden Bridge, China) and examined under a light microscope (CX43; Olympus, Japan).

### 2.10 TUNEL Staining

Paraffin‐embedded kidney sections were deparaffinised, rehydrated and treated with proteinase K (20 μg/mL) for 30 min at room temperature, followed by incubation with TUNEL detection solution (#C1090; Beyotime, China) at 37°C for 2 h. Nuclei were counterstained with DAPI, and fluorescence images were captured using a fluorescence microscope (BX53; Olympus).

### 2.11 Malondialdehyde (MDA) Assay

MDA levels in kidney tissues were measured using the Elabscience MDA ELISA Kit (#E‐EL‐0060; Elabscience, China). Tissues were homogenised in ice‐cold PBS (pH 7.4) and centrifuged at 5000× *g* for 5–10 min at 2°C–8°C. Supernatants were collected and analysed following the manufacturer's protocol. Briefly, 50 μL of sample standard was added to each well of the antigen‐coated plate, followed by biotinylated detection antibody and HRP‐conjugated avidin (both diluted 1:100). After incubation with TMB substrate at 37°C for 15 min, the reaction was terminated, and absorbance was measured at 450 nm. MDA concentrations were calculated based on a standard curve.

### 2.12 Catalase (CAT) Activity

CAT activity was measured using the Elabscience Catalase Activity Assay Kit (#E‐BC‐K031‐M; Elabscience). Kidney tissues were homogenized in 0.01 M PBS (pH 7.4) and centrifuged at 10,000× *g* for 10 min at 4°C. Supernatants were incubated with assay reagents at 37°C. Absorbance was measured at 450 nm, and CAT activity (U/mg protein) was determined based on a standard curve.

### 2.13 Superoxide Dismutase (SOD) Activity

SOD activity was measured using a commercial SOD assay kit (#A001‐3; Nanjing Jiancheng Bioengineering Institute, China). Kidney tissues were homogenised in 0.9% NaCl solution and centrifuged at 2500–3000 rpm for 10 min at 4°C. Supernatants were incubated with enzyme and substrate solutions at 37°C for 20 min. Absorbance was measured at 450 nm, and SOD activity (U/mg protein) was calculated based on the inhibition rate of the reaction.

### 2.14 Measurement of ROS in Kidney Tissues

Total intracellular ROS levels in kidney tissues were measured using a Reactive Oxygen Species Assay Kit (#S0033S, Beyotime, Shanghai, China). Briefly, kidney tissues were homogenised in ice‐cold PBS (1:9, w/v) and centrifuged at 5000× *g* for 10 min at 4°C. The resulting pellet was resuspended in PBS containing 10 μM DCFH‐DA and incubated at 37°C for 60 min, with intermittent mixing. After incubation, the cell suspension was centrifuged at 1000× *g* for 5 min, and the pellet was washed once with PBS to remove excess probe. The final pellet was resuspended in PBS, and the fluorescence intensity was measured using a multi‐mode microplate reader (SuperMax3100; Flash, Shanghai, China) at an excitation wavelength of 488 nm and an emission wavelength of 525 nm.

### 2.15 Dihydroethidium (DHE) Staining

ROS levels in renal tissues were assessed by DHE staining of frozen sections. Fresh kidney samples were embedded in OCT compound, snap‐frozen and sectioned using a cryostat (CM1950; Leica). Sections were air‐dried at room temperature for 5 min and incubated with 1× washing solution for 10 min. After removal of the wash buffer, sections were covered with freshly prepared DHE working solution (1:300 dilution in PBS; G4817; Solarbio, Beijing, China) and incubated at room temperature in the dark for 1 h. Slides were rinsed three times with PBS (5 min each), counterstained with Hoechst 33342 (C1026; Beyotime) for 30 min, and washed again with PBS. Fluorescence signals were visualised under a fluorescence microscope (CKX53; Olympus). ROS‐associated DHE fluorescence was detected at an excitation wavelength of 488–535 nm and an emission wavelength of 610 nm. Nuclear Hoechst staining was observed at 330–380 nm excitation and 420 nm emission. Signal intensities were quantified using ImageJ software.

### 2.16 ELISA for Interleukin‐1 Beta (IL‐1β) and Interleukin‐6 (IL‐6)

The concentrations of IL‐1β and IL‐6 in rat serum samples were quantified using commercial ELISA kits (#E‐EL‐H0149 for IL‐1β; #E‐EL‐H6156 for IL‐6; Elabscience). All procedures were conducted according to the manufacturer's instructions.

### 2.17 Quantitative Reverse Transcription PCR (qRT‐PCR)

Total RNA was extracted from rat kidney tissues or HK‐2 cells using Trizol reagent. mRNA was purified using the mRNA Ultra‐purity extraction kit (#CW0581M; CWBIO, China), and RNA concentration and purity were assessed by measuring the OD260/OD280 ratio using a UV–visible spectrophotometer. cDNA was synthesized using a reverse transcription kit (#R223‐01; Vazyme, China). PCR was performed using a fluorescence‐based PCR instrument under the following conditions: initial denaturation at 95°C for 10 min, followed by 40 cycles of denaturation at 95°C for 10 s, annealing at 58°C for 30 s, and extension at 72°C for 30 s. β‐actin served as the internal reference gene. Relative gene expression was calculated using the 2^−ΔΔCt^ method. Primer sequences are provided in Table S1.

### 2.18 Western Blot Analysis

Kidney tissues and HK‐2 cells from each experimental group were lysed in RIPA buffer. Tissue samples were homogenised, and all lysates were centrifuged at 12,000 rpm for 10 min at 4°C. Supernatants were collected, and total protein concentrations were determined using a BCA protein assay kit (#E‐BC‐K318‐M; Elabscience). Proteins were denatured, separated by SDS‐PAGE (1.5 h), and transferred onto PVDF membranes at 300 mA for 1–2 h. Membranes were blocked with non‐fat milk and incubated overnight at 4°C with the following primary antibodies: mouse anti‐β‐actin (#HC201; 1:2000; TransGen Biotech, Beijing, China), anti‐MLKL (#OM155258; 1:500; OmnimAbs, Alhambra, CA, USA), anti‐GSDMD‐N (#PA5‐115330; 1:1000; Thermo Fisher Scientific, Waltham, MA, USA), anti‐cleaved‐Caspase 3 (#9661; 1:1000; Cell Signalling Technology, Danvers, MA, USA), anti‐ZEB1 (#21544‐1‐AP; 1:1000; Proteintech, Rosemont, IL, USA), and anti‐SENP1 (#25349‐1‐AP; 1:4000; Proteintech). The following day, membranes were incubated for 2 h at room temperature with HRP‐conjugated secondary antibodies: goat anti‐mouse IgG (H+L) (#GB23301; 1:2000; Servicebio, Wuhan, China) or goat anti‐rabbit IgG (H+L) (#GB23303; 1:2000; Servicebio). After thorough washing, protein bands were visualised using enhanced chemiluminescence reagents and imaged with a Tanon‐5200 ultra‐sensitive chemiluminescence system (Tanon Science and Technology, China).

### 2.19 Co‐Immunoprecipitation (Co‐IP) Assay

Co‐IP was conducted using the Pierce Co‐IP kit (#88805; Thermo Fisher Scientific) to examine ZEB1 SUMOylation and ubiquitination. Cells were lysed in cell lysis buffer and gently rotated at 4°C for 30 min. Lysates were centrifuged at 13,000× *g* for 10 min at 4°C, and a portion of the supernatant was retained as the input control. The remaining lysate was incubated with ZEB1 antibody (5 μg) pre‐conjugated to magnetic beads for 30 min at room temperature. An IgG control was included to account for non‐specific binding. The antibody‐bead complex was then mixed with 500 μL of lysate and transferred to a spin column. After overnight incubation at 4°C, the supernatant was discarded, and the column was washed three times with IP buffer to eliminate non‐specific interactions. Bound proteins were eluted with 100 μL of elution buffer and incubated at room temperature for 10 min. The eluates were collected by centrifugation, mixed with loading buffer and DTT, and subjected to Western blotting to detect SUMO1, SUMO2/3 and ubiquitin. The following primary antibodies were used: anti‐SUMO1 (Cat# 10329‐1‐AP, Proteintech, 1:2000 dilution), anti‐SUMO2/3 (Cat# 67154‐1‐Ig, Proteintech, 1:3000 dilution) and anti‐Ubiquitin (Cat# 10201‐2‐AP, Proteintech, 1:2000 dilution).

### 2.20 Statistical Analysis

All experiments were independently repeated at least three times. Data are expressed as mean ± standard deviation (SD). Statistical analyses were performed using SPSS 19.0 (IBM Corp., Armonk, NY, USA). Differences between two groups were assessed using independent sample *t*‐tests. Comparisons among multiple groups were analysed by one‐way ANOVA. A two‐tailed *p* value < 0.05 was considered statistically significant. Graphs were generated using GraphPad Prism 9.0 (GraphPad Software, San Diego, CA, USA).

## 3 Results

### 3.1 ZEB1 Is Upregulated in Human AKI and Associated With PANoptosis‐ and SUMOylation‐Related Genes

Analysis of public transcriptomic datasets (GSE53769, GSE139061) revealed significantly elevated ZEB1 mRNA levels in AKI kidney tissues compared to controls (Figure S1A,B). GTEx data confirmed stable ZEB1 expression in normal kidney tissues (Figure S1C,D). In the GSE139061 dataset, ZEB1 expression showed positive correlations with PANoptosis‐related genes MLKL (*r* = 0.282, *p* = 0.082), GSDMD (*r* = 0.262, *p* = 0.108) and CASP3 (*r* = 0.180, *p* = 0.273; Figure S1E–H). Negative correlations were observed with SUMOylation‐related genes, including SUMO1 (*r* = −0.247, *p* = 0.130), SUMO4 (*r* = −0.312, *p* = 0.053) and SENP7 (*r* = −0.224, *p* = 0.171; Figure S1E–H). GENEMANIA network analysis indicated functional connectivity between ZEB1, PANoptosis caspases and SUMOylation components, showing direct links to GSDME and SUMO1 (Figure S1I,J). These findings collectively suggest that ZEB1 contributes to PANoptotic signalling and may be regulated via SUMOylation‐associated pathways in human AKI.

### 3.2 Burn‐Induced AKI Triggers Oxidative Stress, PANoptosis and ZEB1 SUMOylation in Renal Tissues

**FIGURE 1:**
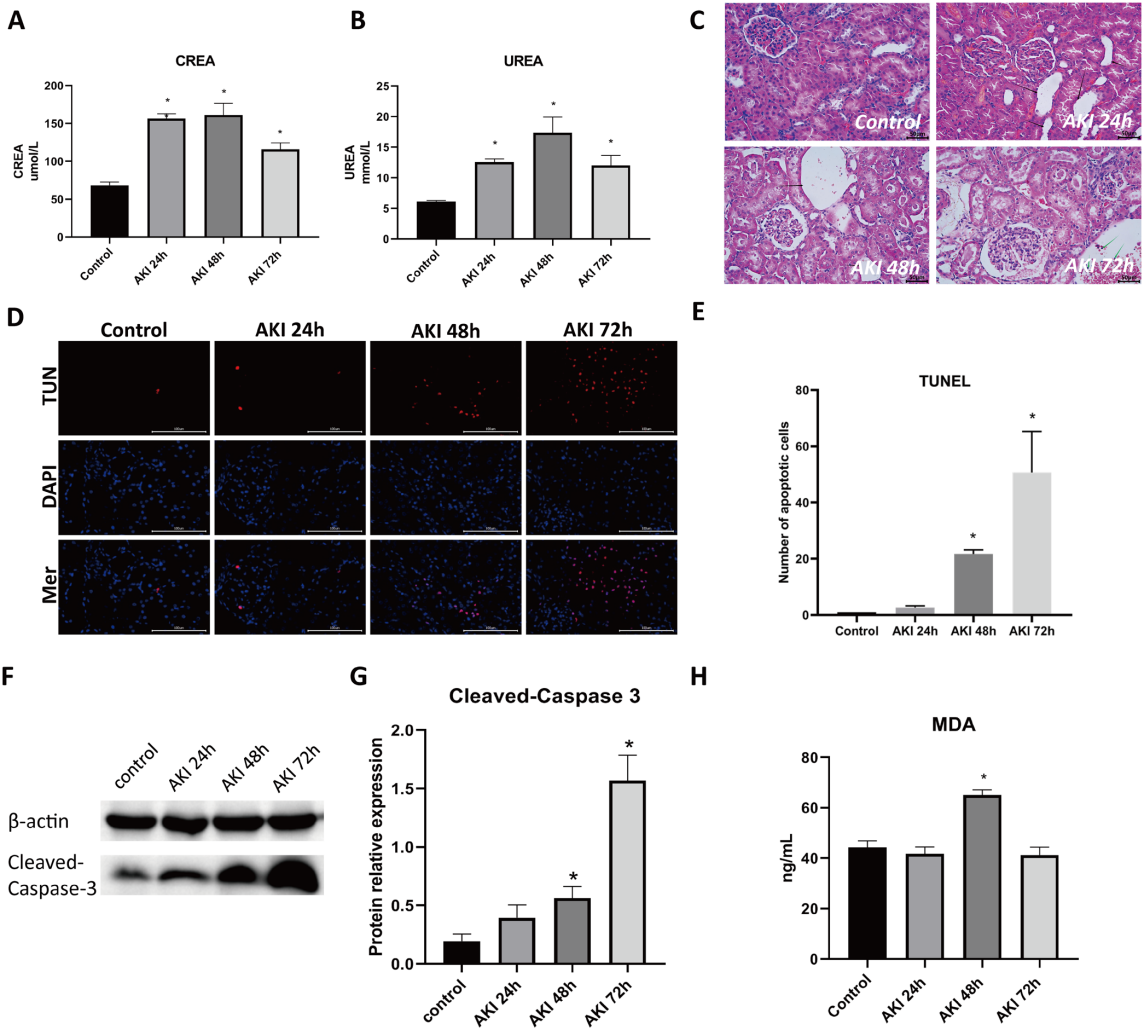
Renal dysfunction, histopathological injury, apoptosis and oxidative stress in burn‐induced acute kidney injury (AKI) model. (A) Plasma creatinine (CREA) levels in control and AKI rats at 24, 48 and 72 h post‐injury. (B) Plasma urea levels in control and AKI rats at 24, 48 and 72 h post‐injury. (C) Representative haematoxylin and eosin (H&E)‐stained kidney sections showing progressive tubular and glomerular injury in AKI groups compared to controls. (D, E) TUNEL staining and quantification of apoptotic cells in kidney tissues from control and AKI rats at 24, 48 and 72 h post‐injury. (F) Western blot analysis of cleaved Caspase‐3 expression in kidney tissues from control and AKI rats at 24, 48 and 72 h post‐injury. (G) Densitometric quantification of cleaved Caspase‐3. (H) Malondialdehyde (MDA) levels indicating lipid peroxidation. *p < 0.05 versus control group.

In the rat burn‐induced AKI model, plasma creatinine and urea levels, indicative of impaired glomerular filtration, were significantly elevated from 24 to 72 h, peaking at 48 h (Figure 1A,B). H&E staining revealed progressive histopathological damage, including tubular dilation and epithelial detachment at 48 h and glomerular atrophy with inflammation at 72 h (Figure 1C). These findings confirm the successful induction of AKI and identify 48 h as a critical window for mechanistic investigation.

To explore underlying mechanisms, we evaluated apoptosis and oxidative stress. TUNEL staining showed a marked increase in apoptotic cells at 48 and 72 h (Figure 1D,E), accompanied by increased cleaved Caspase‐3 expression (Figure 1F,G). MDA levels were significantly elevated at 48 h (Figure 1H), whereas antioxidant enzyme activities (SOD and CAT) declined (Figure S2A,B). DHE staining showed increased ROS accumulation in renal tissues at 24 h, peak levels at 48 h and persistent elevation at 72 h in burn‐induced AKI compared with controls (Figure S2C–E). These results indicate oxidative stress during burn‐induced AKI.

**FIGURE 2:**
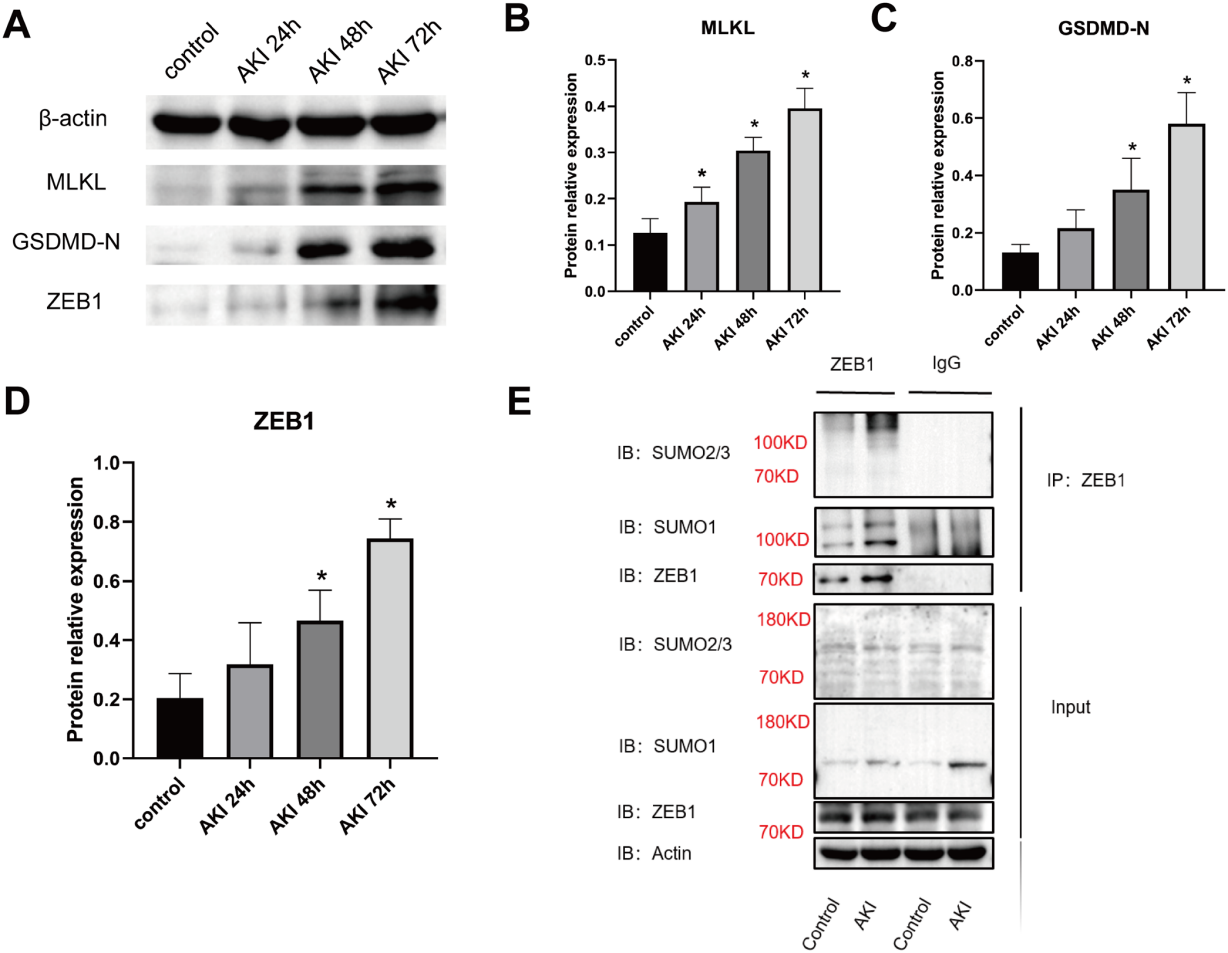
Expression of PANoptosis‐related proteins and ZEB1 SUMOylation in burn‐induced AKI. (A) Western blot analysis of MLKL, GSDMD‐N and ZEB1 in kidney tissues from control and AKI rats at 24, 48 and 72 h post‐injury. (B–D) Densitometric quantification of MLKL (B), GSDMD‐N (C) and ZEB1 (D) relative to β‐actin. (E) Co‐immunoprecipitation (Co‐IP) of ZEB1 followed by immunoblotting with SUMO1 and SUMO2/3 antibodies in control and AKI kidney tissues. Input lanes show total levels of SUMO1, SUMO2/3, ZEB1 and β‐actin. *p < 0.05 versus control group.

Given the role of inflammatory cell death in AKI, we next examined markers of PANoptosis. Western blot analysis showed time‐dependent increases in MLKL and GSDMD‐N from 24 to 72 h (Figure 2A–C), supporting the activation of necroptosis and pyroptosis. As noted above, cleaved Caspase‐3 was also upregulated during this period (Figure 1F,G), indicating concurrent apoptotic activation. ZEB1 expression was significantly upregulated in AKI tissues (Figure 2A,D). Co‐IP demonstrated increased ZEB1 conjugation with SUMO1 and SUMO2/3 (Figure 2E), suggesting that ZEB1 SUMOylation may contribute to PANoptotic signalling in burn‐induced AKI.

### 3.3 H/R Induces Oxidative Stress, Inflammatory Responses and PANoptotic Cell Death in Renal Tubular Epithelial Cells

**FIGURE 3:**
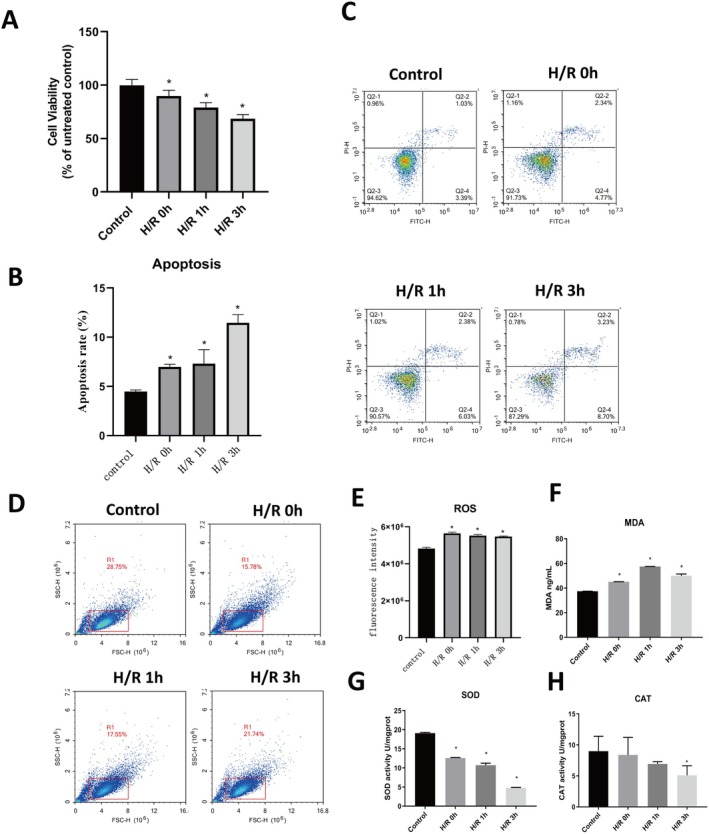
Hypoxia‐reoxygenation (H/R) induces time‐dependent apoptosis and oxidative stress in HK‐2 cells. (A) Cell viability measured by CCK‐8 assay at 0, 1 and 3 h of reoxygenation. (B, C) Quantification and representative flow cytometry plots of apoptotic cells. (D, E) Flow cytometric analysis and quantification of intracellular ROS levels. (F–H) Measurement of malondialdehyde (MDA) content, superoxide dismutase (SOD) activity and catalase (CAT) activity at corresponding time points. *p < 0.05 versus control group.

To model renal epithelial injury in vitro, HK‐2 cells were exposed to H/R for 0, 1, or 3 h. H/R treatment of HK‐2 cells resulted in time‐dependent declines in cell viability (Figure 3A) and increases in apoptosis (Figure 3B,C). Intracellular ROS levels were elevated at all H/R time points (Figure 3D,E), accompanied by increased MDA (peaking at 1 h; Figure 3F) and decreased SOD/CAT activities (Figure 3G,H). Additionally, IL‐1β and IL‐6 secretion increased in a time‐dependent manner (Figure S3), indicating sustained inflammatory activation. Analysis of apoptosis, necroptosis and pyroptosis markers showed progressive increases in Caspase‐3 mRNA/cleaved protein (Figure S4A,D,E), delayed MLKL protein upregulation (Figure S4B,D,F) and consistent increases in GSDMD mRNA/GSDMD‐N protein (Figure S4C,D,G). These results indicate that H/R triggers a coordinated activation of apoptosis, necroptosis and pyroptosis, consistent with PANoptosis.

### 3.4 H/R Promotes ZEB1 SUMOylation and Suppresses Its Ubiquitination in HK‐2 Cells

**FIGURE 4:**
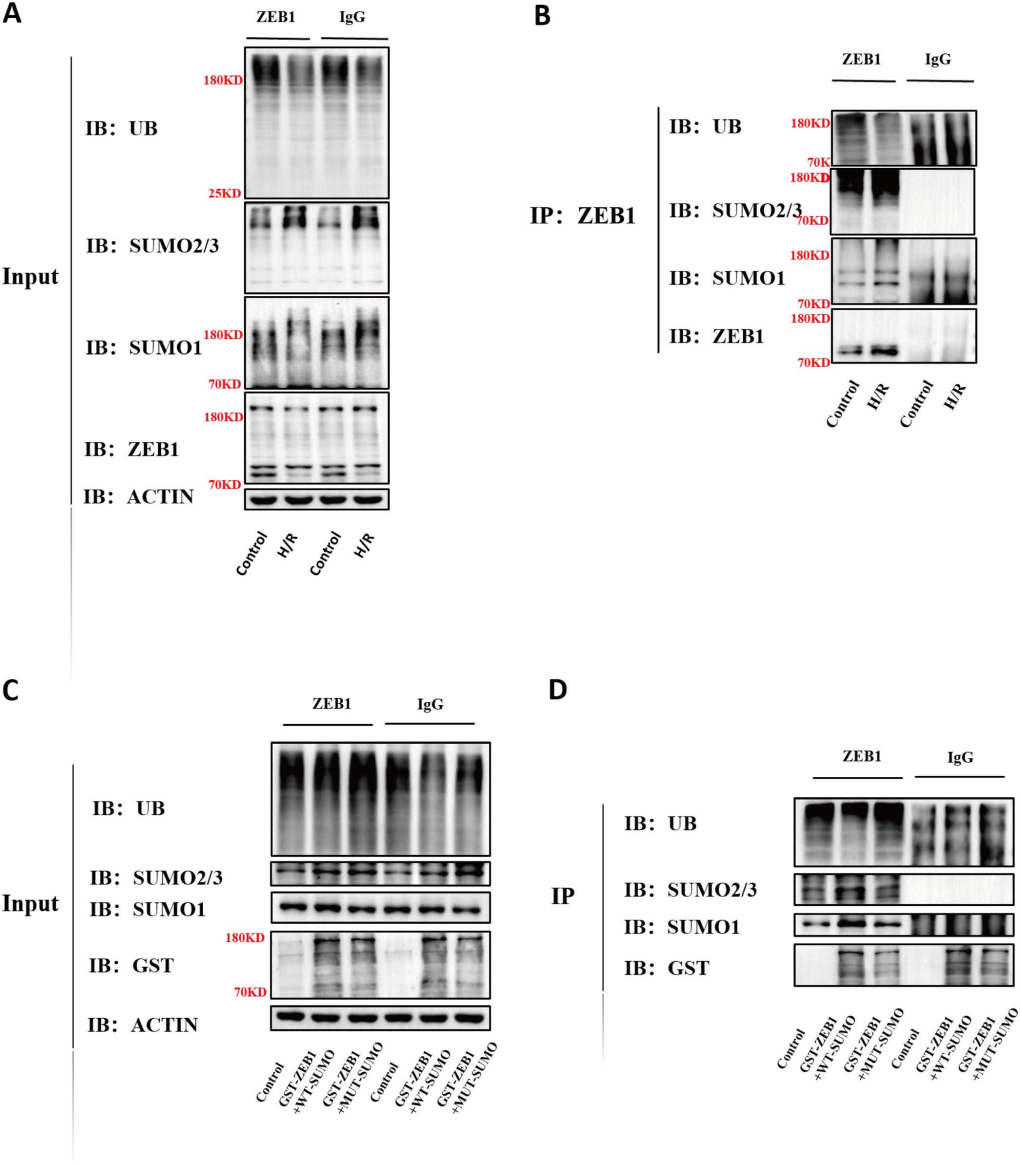
H/R promotes ZEB1 SUMOylation and reduces its ubiquitination. (A, B) Co‐IP and immunoblot analysis of ZEB1 SUMOylation and ubiquitination in HK‐2 cells under normoxic or H/R conditions. ZEB1 was immunoprecipitated and probed with antibodies against SUMO1, SUMO2/3 and ubiquitin. (C, D) In vitro SUMOylation and ubiquitination assays using GST‐tagged ZEB1 co‐expressed with wild‐type SUMO (WT‐SUMO) or conjugation‐deficient mutant SUMO (MUT‐SUMO), followed by immunoblotting. IB, immunoblotting; IP, immunoprecipitation.

To investigate whether ZEB1 post‐translational modification contributes to H/R‐induced PANoptosis, we examined its SUMOylation and ubiquitination status. Immunoprecipitation of ZEB1 from H/R‐treated HK‐2 cells followed by immunoblotting revealed markedly enhanced ZEB1 SUMOylation (increased SUMO1/SUMO2/3 signals) and reduced ZEB1 ubiquitination (Figure 4A,B). Co‐expression of GST‐ZEB1 with wild‐type SUMO increased SUMO1/2/3 association and reduced ubiquitination, whereas a SUMO conjugation‐deficient mutant (MUT‐SUMO) failed to induce these changes (Figure 4C,D). These findings suggest that H/R promotes ZEB1 SUMOylation in a SUMO‐dependent manner, which may stabilize ZEB1 by limiting its ubiquitin‐mediated degradation.

### 3.5 SENP1 Overexpression Decreases ZEB1 Expression, Increases Global Ubiquitination and Reduces ZEB1 SUMOylation Under H/R Conditions

**FIGURE 5:**
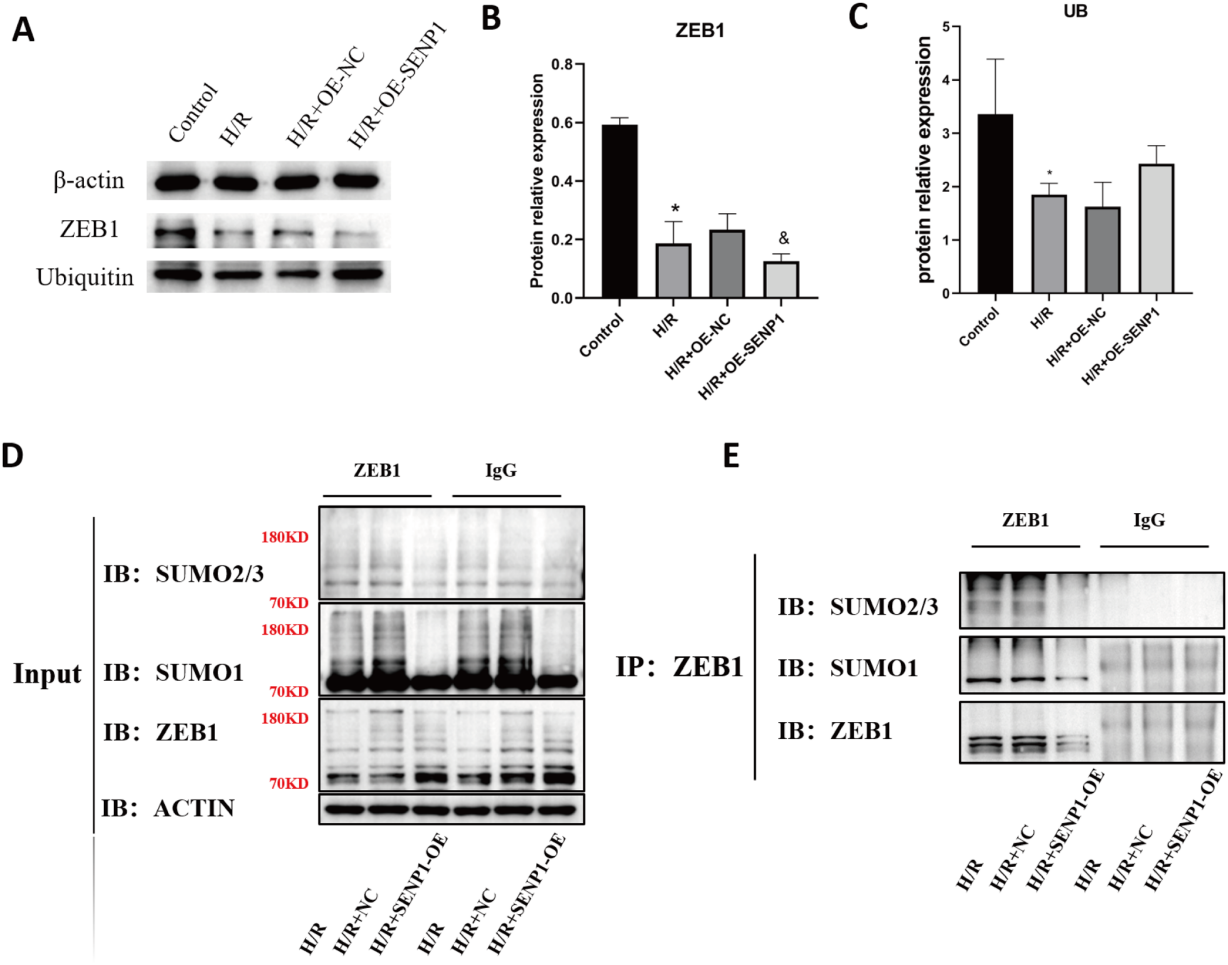
SENP1 overexpression is associated with decreased ZEB1 expression, increased global ubiquitination and reduced SUMOylation under H/R conditions. (A) Western blot analysis of ZEB1 and ubiquitin (UB) levels in control or SENP1‐overexpressing HK‐2 cells under normoxic and H/R conditions. (B, C) Quantification of ZEB1 (B) and total ubiquitin (C) protein levels normalised to β‐actin. (D, E) Co‐IP of ZEB1 followed by immunoblotting for SUMO2/3 and SUMO1 to assess ZEB1 SUMOylation in the presence or absence of SENP1 overexpression under H/R. *p < 0.05 versus the Control group; ^&^ *p* < 0.05 versus the H/R+OE‐NC group.

To further elucidate the regulatory mechanism of ZEB1 SUMOylation, we investigated the role of SENP1, a SUMO‐specific protease. SENP1 mRNA was downregulated by H/R treatment (Figure S5A). SENP1 overexpression was confirmed at mRNA and protein levels (Figure S5B–D). Under H/R, SENP1 overexpression further reduced ZEB1 protein levels (Figure 5A,B). Concurrently, H/R reduced global ubiquitination levels, whereas SENP1 overexpression restored ubiquitination to near‐baseline levels (Figure 5A,C), suggesting that SENP1 promotes ZEB1 degradation by reversing its SUMOylation and facilitating ubiquitin‐mediated turnover. Co‐immunoprecipitation confirmed that SENP1 overexpression decreased ZEB1 SUMOylation, as indicated by reduced SUMO1 and SUMO2/3 signals (Figure 5D,E). These findings suggest that SENP1 facilitates ZEB1 degradation under H/R by enhancing its ubiquitination and diminishing its SUMOylation.

### 3.6 Inhibition of ZEB1 SUMOylation or Knockdown of ZEB1 Attenuates H/R‐Induced Apoptosis, Oxidative Stress and Inflammation in HK‐2 Cells

**FIGURE 6:**
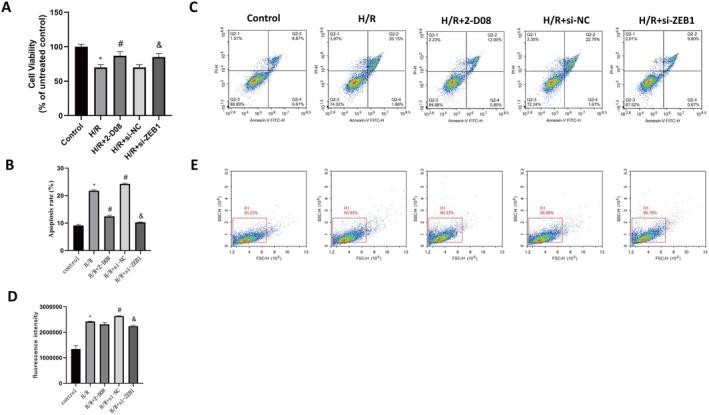
Inhibition of ZEB1 SUMOylation or knockdown of ZEB1 mitigates H/R‐induced cell injury in HK‐2 cells. (A) CCK‐8 assay showing cell viability in H/R‐treated cells subjected to SUMOylation inhibition (2‐D08) or ZEB1 knockdown. (B) Quantification of apoptotic cell percentages assessed by flow cytometry. (C) Representative Annexin V/PI flow cytometry dot plots illustrating apoptotic populations in each treatment group. (D) Intracellular ROS levels measured by fluorescence intensity in cells subjected to the indicated treatments. (E) Representative flow cytometry plots showing ROS‐positive populations. *p < 0.05 versus Control, ^#^ *p* < 0.05 versus H/R, ^&^ *p* < 0.05 versus H/*R* + si‐NC.

Having established that SENP1 regulates ZEB1 SUMOylation under H/R conditions, we next examined the functional consequences of ZEB1 SUMOylation during H/R injury. ZEB1 knockdown efficiency was validated using three siRNAs (Figure S6A–C). Pharmacological inhibition of SUMOylation (2‐D08) or ZEB1 knockdown significantly improved cell viability (Figure 6A) and attenuated apoptosis (Figure 6B,C) under H/R. ZEB1 knockdown markedly reduced ROS levels, while 2‐D08 had a limited effect (Figure 6D,E). Both treatments suppressed H/R‐induced IL‐1β and IL‐6 production (Figure S7). These findings indicate that ZEB1 SUMOylation contributes to H/R‐induced apoptosis, oxidative stress and inflammation, which are integral components of PANoptosis.

### 3.7 Inhibition of ZEB1 SUMOylation Dampens PANoptosis Effector Activation Under H/R Conditions

**FIGURE 7:**
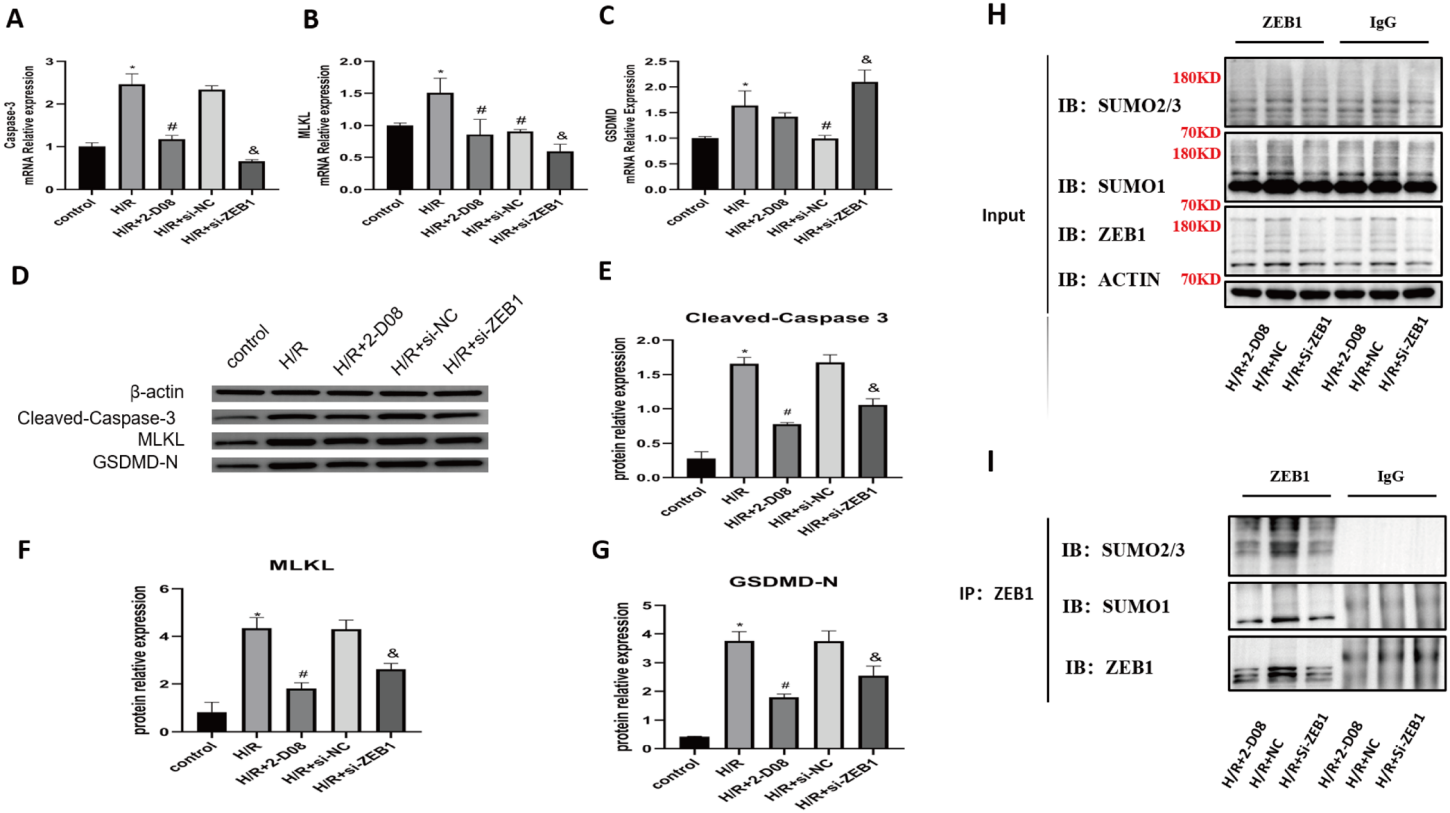
ZEB1 SUMOylation promotes PANoptosis‐related gene expression and is reduced by 2‐D08 or ZEB1 knockdown under H/R conditions. (A–C) Quantification of Caspase‐3 (A), MLKL (B) and GSDMD (C) mRNA levels in control and H/R‐treated HK‐2 cells, with or without 2‐D08 treatment or ZEB1 knockdown. (D) Western blot analysis of cleaved Caspase‐3, MLKL and GSDMD‐N protein expression. (E–G) Quantification of protein expression from panel D for cleaved Caspase‐3 (E), MLKL (F) and GSDMD‐N (G). Data are normalised to β‐actin and presented as mean ± SD. *p < 0.05 versus control, ^#^ *p* < 0.05 versus H/R, ^&^ *p* < 0.05 versus H/*R* + si‐NC. (H) Input and Co‐IP western blot showing total and immunoprecipitated levels of SUMO2/3, SUMO1 and ZEB1 following 2‐D08 treatment or ZEB1 knockdown. (I) Co‐IP of ZEB1 with SUMO2/3 and SUMO1 in cells under different treatment conditions. ZEB1 immunoprecipitates were analysed by immunoblotting.

To determine whether ZEB1 SUMOylation is necessary for the execution of PANoptosis, we assessed the expression of core effectors at both mRNA and protein levels. H/R increased mRNA levels of Caspase‐3 and MLKL, which were attenuated by 2‐D08 or ZEB1 knockdown. GSDMD mRNA was further elevated by ZEB1 knockdown (Figure 7A–C), despite a concurrent reduction in GSDMD‐N protein levels (Figure 7D,G), indicating a potential mRNA–protein discrepancy. Protein levels of cleaved Caspase‐3, MLKL and GSDMD‐N were upregulated by H/R and partially suppressed by both interventions (Figure 7D–G). Co‐IP confirmed reduced ZEB1 SUMOylation in intervention groups (Figure 7H,I), indicating that ZEB1 SUMOylation facilitates PANoptotic effector activation in H/R‐induced injury.

## 4 Discussion

Burn‐induced AKI is a significant complication negatively impacting patient prognosis, driven by factors like shock, toxins, infection and medications that impair renal function [1, 22]. This rapid dysfunction within hours to days results in compromised waste filtration. Our study confirmed significant renal dysfunction and progressive histopathological damage, peaking at 48 h post‐injury in the burn AKI rat model, aligning with the rapid onset and progression of AKI and providing an optimal time window for investigation.

The pathophysiology of burn‐related AKI is complex, involving ischemia, inflammation and immune dysregulation [17]. Hypovolemia and reduced renal perfusion cause ischemic injury, while inflammatory mediators and oxidative stress exacerbate damage. Consistent with this, our study showed increased oxidative stress (elevated MDA, reduced SOD/CAT) and apoptosis (TUNEL staining) in renal tissues following AKI. Crucially, we observed increased expression of PANoptosis markers (cleaved Caspase‐3, MLKL, GSDMD‐N), supporting the activation of multiple inflammatory cell death pathways in the burn model. This validates our model's suitability for studying burn‐induced renal injury mechanisms.

During H/R, excessive ROS and reactive nitrogen species are generated, leading to oxidative damage of membrane lipids, proteins and DNA, ultimately resulting in structural and functional deterioration of renal tubular cells [23]. We found H/R induced time‐dependent cytotoxicity, increased ROS, decreased antioxidants and elevated inflammatory cytokines (IL‐1β, IL‐6), consistent with sustained inflammation. Importantly, H/R activated apoptosis, necroptosis and pyroptosis markers (Caspase‐3, MLKL, GSDMD), supporting a coordinated PANoptotic response [24]. The observed discrepancy between GSDMD mRNA and protein levels in H/R cells warrants further investigation into post‐transcriptional regulation or cleavage‐dependent activation, highlighting the complexity of PANoptotic control.

ZEB1, a transcription factor, plays a crucial role in cell differentiation, apoptosis and inflammatory responses. It is involved in maintaining tissue homeostasis, regulating stem cell properties and modulating gene expression in response to environmental stressors [25]. ZEB1 has been implicated in fibrosis‐related disorders, including pulmonary and renal fibrosis [10, 26]. While some studies suggest that ZEB1 downregulation contributes to ROS‐induced apoptosis and cellular senescence [27], others have shown that ZEB1 knockdown attenuates apoptosis and restores cell proliferation in renal epithelial cells [12]. These findings highlight the context‐dependent roles of ZEB1 in cell death regulation. In our burn and H/R‐induced AKI models, we observed that ZEB1 expression and SUMOylation were markedly elevated, correlating with enhanced apoptosis and inflammation. This discrepancy may reflect differences in injury type, cell types, or the post‐translational modification status of ZEB1, such as SUMOylation, which may shift ZEB1 toward a pro‐death phenotype.

SUMOylation is a key post‐translational modification regulating protein localization and transcription factor activity, often competing with ubiquitination to affect stability [28, 29]. Recent studies have highlighted the critical role of SUMOylation in various physiological and pathological processes, including cancer, neurodegeneration and metabolic disorders [29, 30, 31]. In AKI, SUMOylation has been shown to regulate key transcription factors involved in oxidative stress and apoptosis [32, 33]. For instance, SUMOylation of p53 exerts a protective effect against tubular cell apoptosis, thereby preserving renal function [34]. Despite this, SUMOylation's role in burn AKI is unclear. Our study identified a functional link between ZEB1 and SUMO1 (STRING database), showing burn AKI significantly enhanced ZEB1 SUMOylation while inhibiting ubiquitination, increasing ZEB1 stability/function. SENP1, a SUMO‐specific protease downregulated by H/R, facilitated ZEB1 ubiquitination/degradation by removing SUMO, thus attenuating its effects. This highlights the dynamic SUMOylation–ubiquitination interplay in ZEB1 regulation during AKI. SENP1 overexpression in our model restored global ubiquitination and reduced ZEB1 SUMOylation/protein, suggesting its role in balancing survival and PANoptosis. Notably, SENP1 has also been shown to protect against cisplatin‐induced AKI by limiting apoptosis through stabilization of HIF‐1α, a nephroprotective transcription factor [35]. Our findings underscore SENP1 as a broader regulator of stress‐responsive transcription factors and identify the SENP1–ZEB1 axis as a potential checkpoint for restraining PANoptosis in burn‐induced AKI.

ZEB1 also promotes oxidative stress by suppressing GPX4 [36] and activating GSDME to trigger pyroptosis [13]. To further elucidate the role of ZEB1 in H/R‐induced cell death, we employed pharmacological inhibition of SUMOylation using 2‐D08 and genetic knockdown of ZEB1. Both approaches significantly reduced H/R‐induced PANoptosis and improved cell viability. Moreover, ZEB1 knockdown led to a marked decrease in intracellular ROS levels and pro‐inflammatory cytokine production (IL‐1β and IL‐6), further supporting its role in oxidative stress and inflammation.

Mechanistically, ZEB1 SUMOylation was found to regulate the expression of key PANoptosis mediators, including Caspase‐3, MLKL and GSDMD. While our study demonstrated that SUMOylation enhanced ZEB1 stability by inhibiting its ubiquitination, the precise mechanisms through which SUMOylated‐ZEB1 promotes PANoptosis warrant deeper exploration. One possibility is that SUMOylation facilitates ZEB1‐mediated transcriptional activation of pyroptosis‐related genes such as GSDME [13], thereby enhancing pyroptotic signalling and contributing to PANoptosis. Alternatively, SUMOylation may enable ZEB1 to recruit chromatin‐modifying complexes, such as HDACs [37], thereby altering the epigenetic state of PANoptosis‐related genes. These hypotheses are supported by the observed concordant reduction in PANoptotic effectors upon ZEB1 SUMOylation inhibition. Given its dual role in stabilising ZEB1 and enhancing its pro‐PANoptotic activity, SUMOylation emerges as a critical regulatory node linking stress signalling to cell death execution. Importantly, targeting the SUMOylation status of ZEB1, rather than ZEB1 itself, may offer a more selective therapeutic strategy to modulate PANoptosis without broadly impairing its homeostatic functions. This highlights the potential of ZEB1 SUMOylation as both a mechanistic checkpoint and a tractable pharmacologic target in burn‐induced AKI.

Oxidative stress and inflammation are not only hallmarks of burn‐induced AKI but also serve as upstream triggers of PANoptotic cell death [38]. Upon burn or H/R injury, rapid generation of ROS and pro‐inflammatory cytokines initiates stress‐responsive signalling cascades [39]. Our findings suggest that ZEB1 SUMOylation may act as a central regulatory switch, integrating these upstream stress signals and enabling activation of PANoptosis. Notably, PANoptosis is a highly inflammatory form of cell death that exacerbates tissue damage by releasing damage‐associated molecular patterns (DAMPs) and inflammatory mediators [40]. This creates a vicious feedback loop, in which PANoptotic execution further amplifies oxidative stress and inflammation, thereby reinforcing the cycle of injury. Thus, targeting ZEB1 SUMOylation not only inhibits PANoptotic signalling but also disrupts this self‐perpetuating loop, thereby attenuating both primary and secondary damage.

While our study provides new insights, it is important to acknowledge its limitations to guide future research. First, the study was conducted in a rat model and HK‐2 cells, which may not fully recapitulate human AKI pathophysiology. Second, the therapeutic interventions, such as ZEB1 knockdown, were tested in vitro, and their efficacy and safety in vivo remain to be validated. Third, the focus on oxidative stress and apoptosis does not exclude the involvement of other pathways in AKI progression. Additionally, the long‐term effects of ZEB1 SUMOylation inhibition and the potential side effects of SUMOylation inhibitors need to be explored in future studies. Future research should focus on unravelling the molecular mechanisms underlying ZEB1 SUMOylation and its interactions with other post‐translational modifications. Beyond mechanistic validation, research should extend to clinical applications, exploring the feasibility of targeting ZEB1 SUMOylation in burn patients with AKI.

## 5 Conclusions

In summary, this study identifies ZEB1 SUMOylation as a novel regulatory mechanism driving PANoptosis in burn‐induced AKI. By enhancing ZEB1 stability and transcriptional activity, SUMOylation amplifies oxidative stress, inflammatory responses and PANoptosis in renal tubular epithelial cells. Pharmacological or genetic inhibition of this modification attenuates PANoptotic signaling and mitigates renal injury, highlighting ZEB1 SUMOylation as a potential therapeutic checkpoint, offering a new selective approach for modulating aberrant cell death in burn‐related AKI.

## Author Contributions

**Juncheng Zhang:** conceptualization (equal), data curation (equal), formal analysis (equal), writing – original draft (equal), writing – review and editing (equal). **Zhengjie Huang:** conceptualization (equal), data curation (equal), writing – original draft (equal), writing – review and editing (equal). **Qin Lin:** conceptualization (equal), formal analysis (equal), writing – original draft (equal), writing – review and editing (equal). **Hongbin Zhong:** conceptualization (equal), writing – original draft (equal), writing – review and editing (equal). **Weiping Hu:** data curation (equal), writing – original draft (equal), writing – review and editing (equal). **Fengling Zhang:** formal analysis (equal), writing – original draft (equal), writing – review and editing (equal). **Jiyi Huang:** writing – original draft (equal), writing – review and editing (equal).

## Ethics Statement

All animal procedures were approved by the Institutional Animal Care and Use Committee of Jiangxi Zhonghong Boyuan Biotechnology Co. Ltd. (Approval No. LL‐202407050001), and all methods are reported in accordance with ARRIVE guidelines.

## Consent

The authors have nothing to report.

## Conflicts of Interest

The authors declare no conflicts of interest.

## Supporting information


**Figure S1:** Bioinformatic characterisation of ZEB1 expression and its correlation with PANoptosis‐ and SUMOylation‐related genes in human AKI. (A, B) ZEB1 mRNA expression levels in kidney tissue from AKI patients and controls based on GSE53769 (A) and GSE139061 (B). (C, D) Baseline ZEB1 expression across human tissues from the GTEx database (C), stratified by sex (D). (E) Spearman correlation analysis between ZEB1 and PANoptosis‐ or SUMOylation‐related genes in GSE139061, displayed as a lollipop plot with correlation coefficients and *p* values. (F–H) Scatter plots showing positive correlations between ZEB1 and MLKL (F), GSDMD (G) and CASP3 (H) expression in GSE139061. (I, J) GENEMANIA network analysis illustrating ZEB1‐centered functional interactions with PANoptosis‐ and SUMOylation‐related genes (I) and a filtered view showing direct links to GSDME and SUMO1 (J).

**Figure S2:** Antioxidant enzyme activities and reactive oxygen species (ROS) accumulation in burn‐induced AKI. (A, B) Superoxide dismutase (SOD) and catalase (CAT) activities in kidney tissues at 24, 48 and 72 h post‐injury. (C) Quantification of total intracellular ROS levels in kidney tissue homogenates using a DCFH‐DA probe, measured by fluorescence intensity. (D) Quantification of DHE fluorescence intensity expressed as integrated optical density (IOD) per area. (E) Representative images of dihydroethidium (DHE) staining in kidney sections from control and burn‐induced AKI rats at 24 h, 48 h and 72 h. Nuclei were counterstained with DAPI. Scale bar = 100 μm. *p < 0.05 versus control group.

**Figure S3:** H/R increases IL‐1β and IL‐6 expression in HK‐2 cells. (A) ELISA analysis of IL‐1β levels at 0, 1 and 3 h of reoxygenation following hypoxia. (B) ELISA quantification of IL‐6 levels at the same time points. *p < 0.05 versus control group.

**Figure S4:** H/R induces activation of apoptosis, necroptosis and pyroptosis in HK‐2 cells. (A–C) mRNA expression of Caspase‐3 (A), MLKL (B) and GSDMD (C) at 0, 1 and 3 h post‐reoxygenation. (D) Western blot analysis of cleaved Caspase‐3, MLKL and GSDMD‐N protein levels. (E–G) Quantification of cleaved Caspase‐3 (E), MLKL (F) and GSDMD‐N (G) protein expression. All protein levels were normalised to β‐actin. *p < 0.05 versus control group.

**Figure S5:** Analysis of SENP1 expression under H/R conditions and validation of SENP1 overexpression in HK‐2 cells. (A) qRT‐PCR analysis of SENP1 mRNA levels under normoxia and H/R conditions. (B, C) Validation of SENP1 overexpression efficiency at the mRNA level by qRT‐PCR (B) and at the protein level by Western blotting (C). (D) Quantification of SENP1 protein expression normalised to GAPDH. *p < 0.05 versus control; #*p* < 0.05 versus NC.

**Figure S6:** Validation of ZEB1 knockdown efficiency in HK‐2 cells. (A) qRT‐PCR analysis of ZEB1 mRNA expression following transfection with three different ZEB1‐targeting siRNAs or a negative control (NC). (B) Western blot analysis of ZEB1 protein levels in the same conditions. (C) Densitometric quantification of ZEB1 protein expression normalised to GAPDH. Data are presented as mean ± SD (*n* = 3). *p < 0.05 versus control.

**Figure S7:** Inflammatory cytokine levels in HK‐2 cells under H/R with ZEB1 knockdown or SUMOylation inhibition. (A) ELISA quantification of IL‐1β levels in culture supernatants from cells under five conditions: Control, H/R, H/R + 2‐D08, H/R + si‐NC and H/R + si‐ZEB1. (B) ELISA quantification of IL‐6 levels under the same treatment conditions. Data are presented as mean ± SD (*n* = 3). *p < 0.05 versus control; #*p* < 0.05 versus H/R; &*p* < 0.05 versus H/R + si‐NC.

**Table S1:** Primers used in qPCR.

## Data Availability

All data generated or analysed during this study are included in this published article.
