# Supplementary figures and images for: SUMOylation of ZEB1 Modulates PANoptosis in Burn‐Induced Early Acute Kidney Injury
Source: J Cell Mol Med. 2025 Oct 22;29(20):e70865. doi: 10.1111/jcmm.70865 (PMC12544699; doi:10.1111/jcmm.70865)

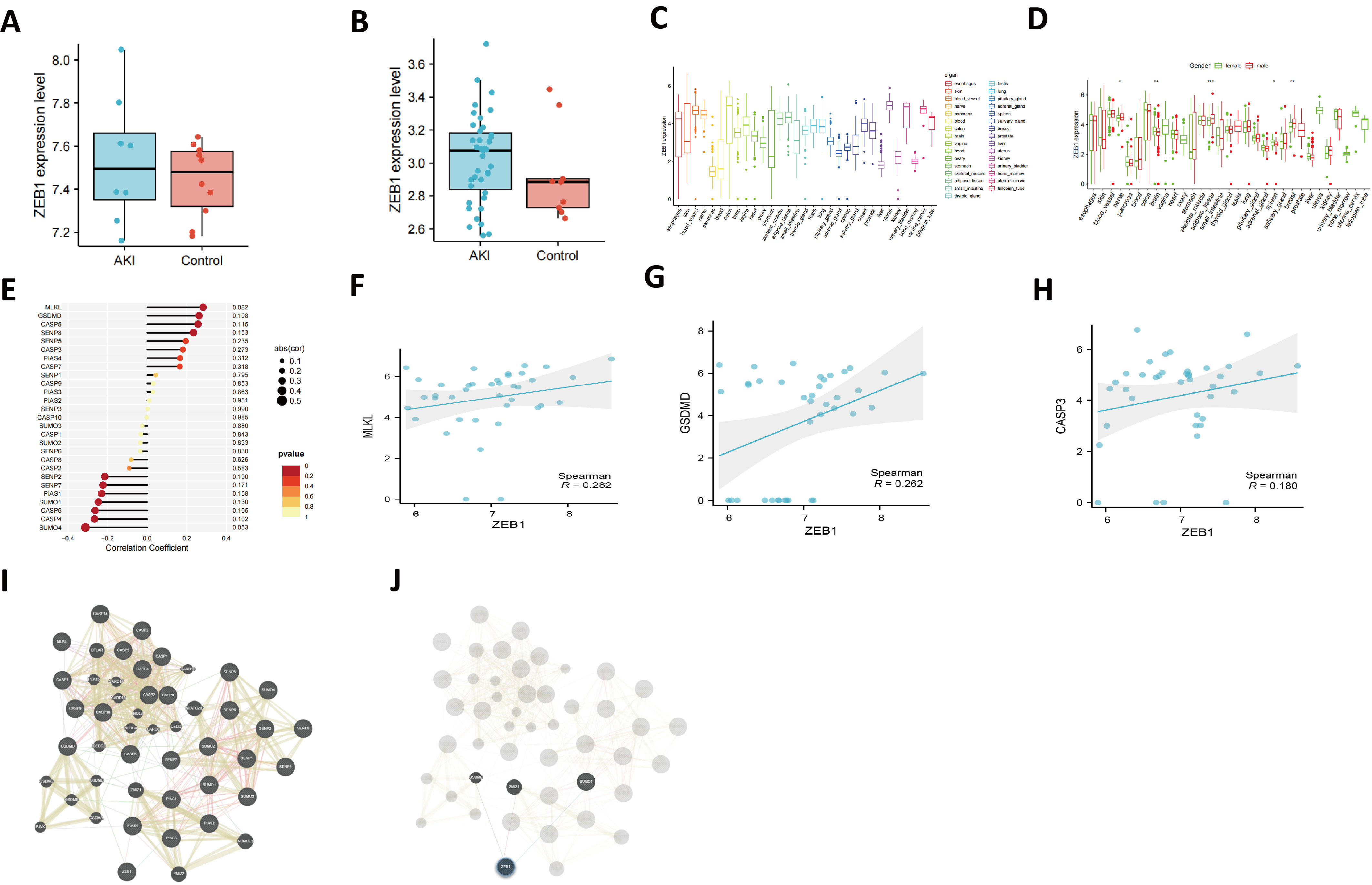

Supplement: Supplementary file 1 — Figure S1: Bioinformatic characterisation of ZEB1 expression and its correlation with PANoptosis‐ and SUMOylation‐related genes in human AKI. (A, B) ZEB1 mRNA expression levels in kidney tissue from AKI patients and controls based on GSE53769 (A) and GSE139061 (B). (C, D) Baseline ZEB1 expression across human tissues from the GTEx database (C), stratified by sex (D). (E) Spearman correlation analysis between ZEB1 and PANoptosis‐ or SUMOylation‐related genes in GSE139061, displayed as a lollipop plot with correlation coefficients and p values. (F–H) Scatter plots showing positive correlations between ZEB1 and MLKL (F), GSDMD (G) and CASP3 (H) expression in GSE139061. (I, J) GENEMANIA network analysis illustrating ZEB1‐centered functional interactions with PANoptosis‐ and SUMOylation‐related genes (I) and a filtered view showing direct links to GSDME and SUMO1 (J). [file JCMM-29-e70865-s001.jpg]

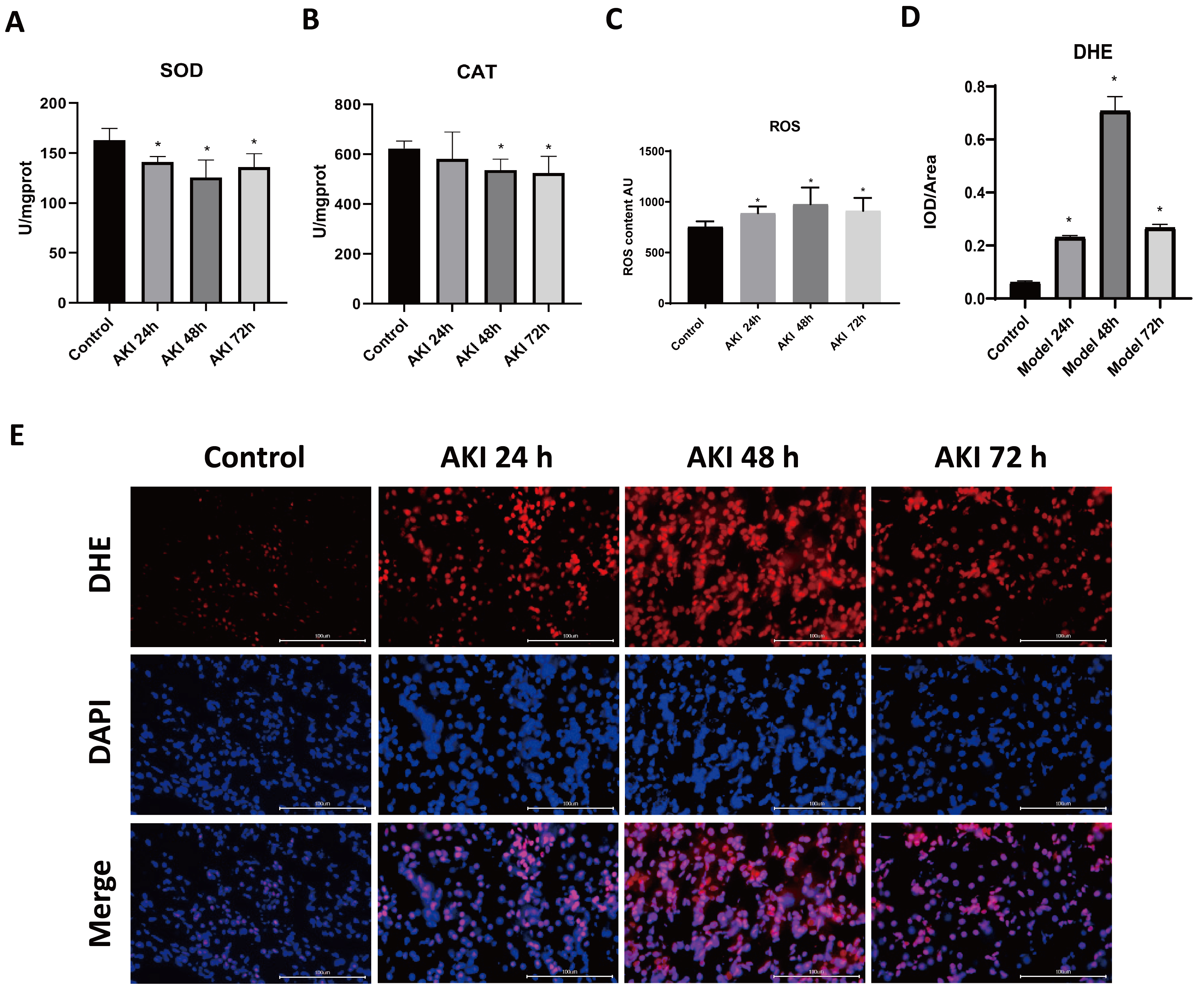

Supplement: Supplementary file 2 — Figure S2: Antioxidant enzyme activities and reactive oxygen species (ROS) accumulation in burn‐induced AKI. (A, B) Superoxide dismutase (SOD) and catalase (CAT) activities in kidney tissues at 24, 48 and 72 h post‐injury. (C) Quantification of total intracellular ROS levels in kidney tissue homogenates using a DCFH‐DA probe, measured by fluorescence intensity. (D) Quantification of DHE fluorescence intensity expressed as integrated optical density (IOD) per area. (E) Representative images of dihydroethidium (DHE) staining in kidney sections from control and burn‐induced AKI rats at 24 h, 48 h and 72 h. Nuclei were counterstained with DAPI. Scale bar = 100 μm. *p < 0.05 versus control group. [file JCMM-29-e70865-s002.jpg]

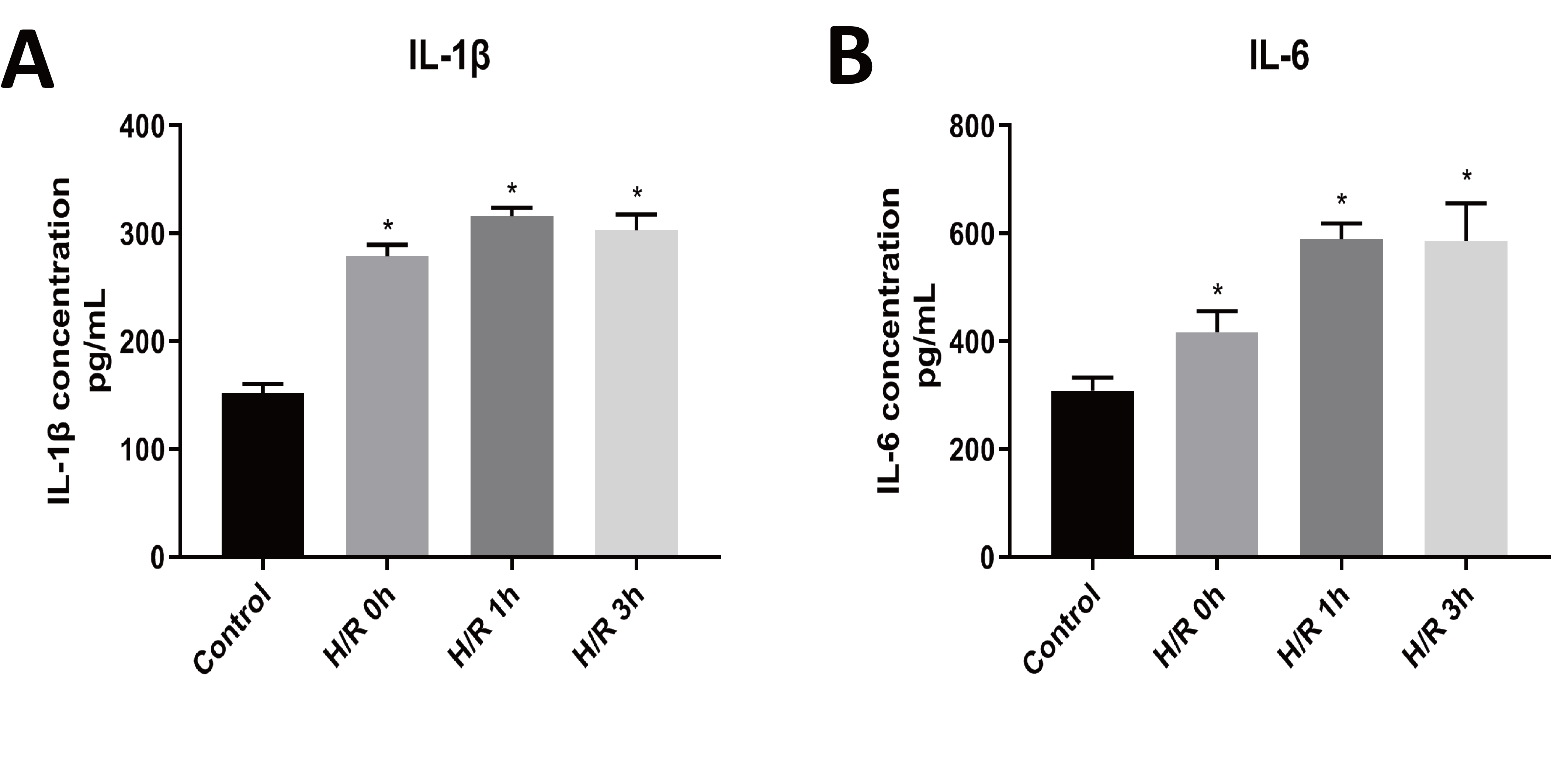

Supplement: Supplementary file 3 — Figure S3: H/R increases IL‐1β and IL‐6 expression in HK‐2 cells. (A) ELISA analysis of IL‐1β levels at 0, 1 and 3 h of reoxygenation following hypoxia. (B) ELISA quantification of IL‐6 levels at the same time points. *p < 0.05 versus control group. [file JCMM-29-e70865-s006.jpg]

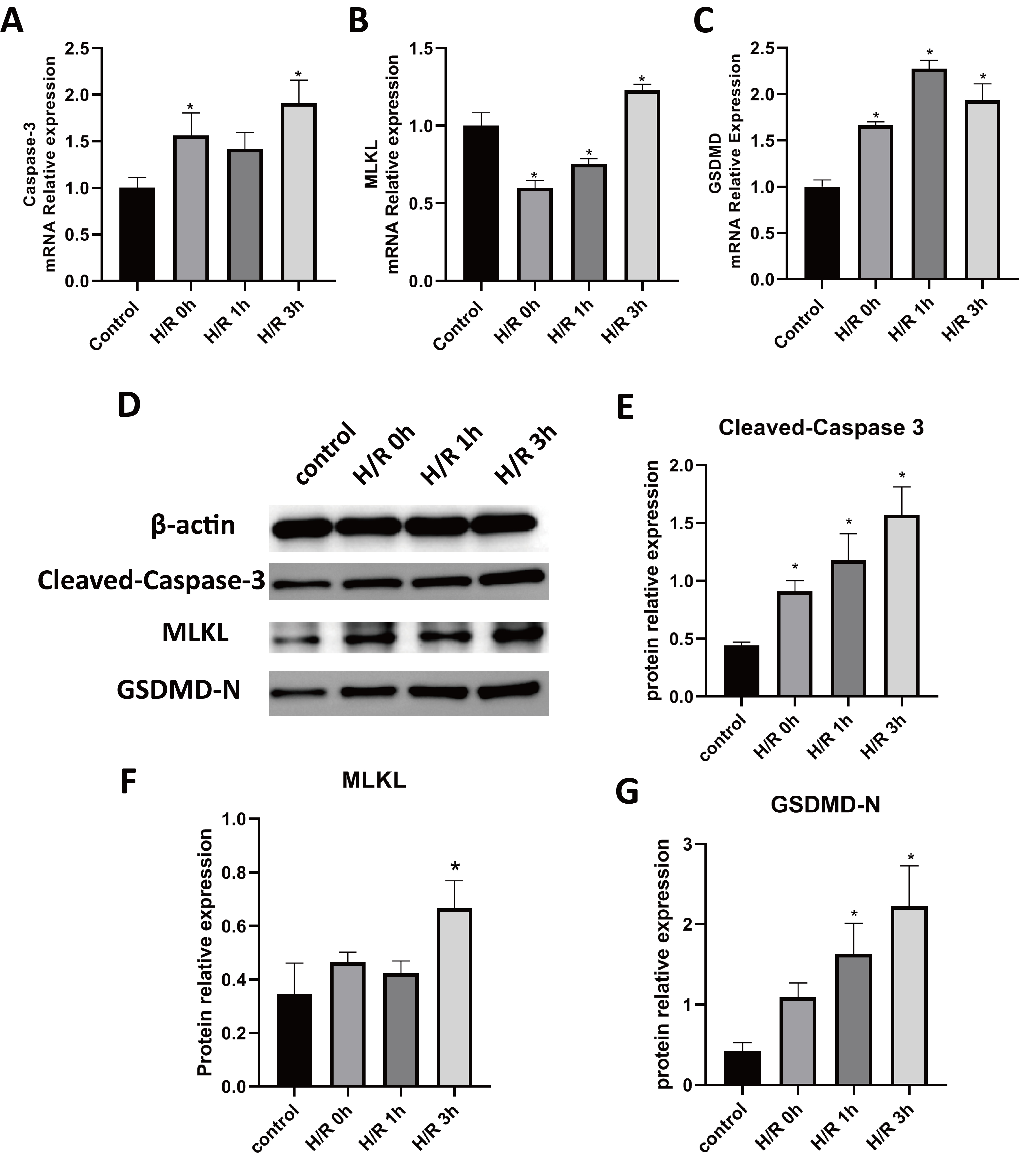

Supplement: Supplementary file 4 — Figure S4: H/R induces activation of apoptosis, necroptosis and pyroptosis in HK‐2 cells. (A–C) mRNA expression of Caspase‐3 (A), MLKL (B) and GSDMD (C) at 0, 1 and 3 h post‐reoxygenation. (D) Western blot analysis of cleaved Caspase‐3, MLKL and GSDMD‐N protein levels. (E–G) Quantification of cleaved Caspase‐3 (E), MLKL (F) and GSDMD‐N (G) protein expression. All protein levels were normalised to β‐actin. *p < 0.05 versus control group. [file JCMM-29-e70865-s004.jpg]

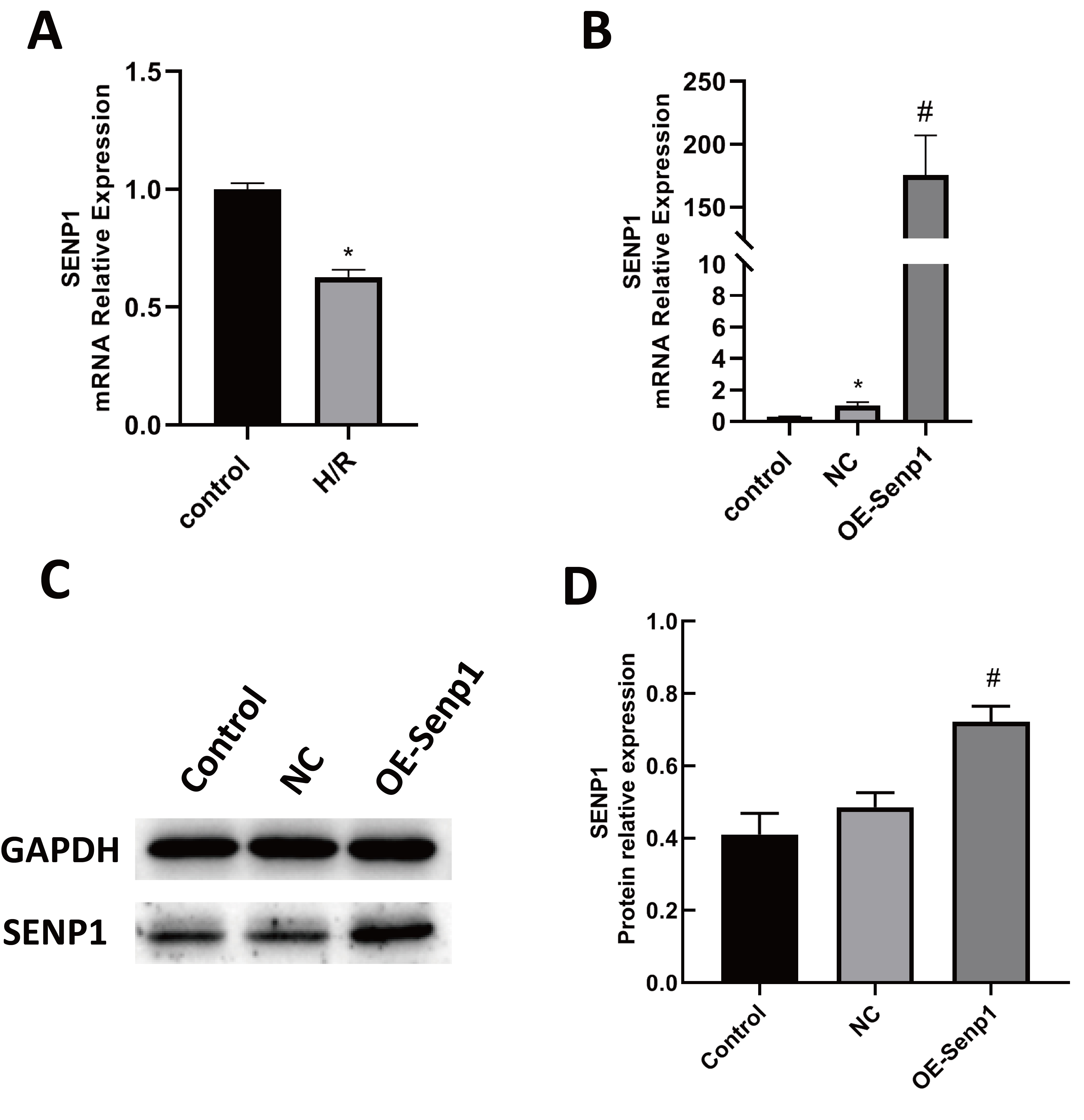

Supplement: Supplementary file 5 — Figure S5: Analysis of SENP1 expression under H/R conditions and validation of SENP1 overexpression in HK‐2 cells. (A) qRT‐PCR analysis of SENP1 mRNA levels under normoxia and H/R conditions. (B, C) Validation of SENP1 overexpression efficiency at the mRNA level by qRT‐PCR (B) and at the protein level by Western blotting (C). (D) Quantification of SENP1 protein expression normalised to GAPDH. *p < 0.05 versus control; #p < 0.05 versus NC. [file JCMM-29-e70865-s008.jpg]

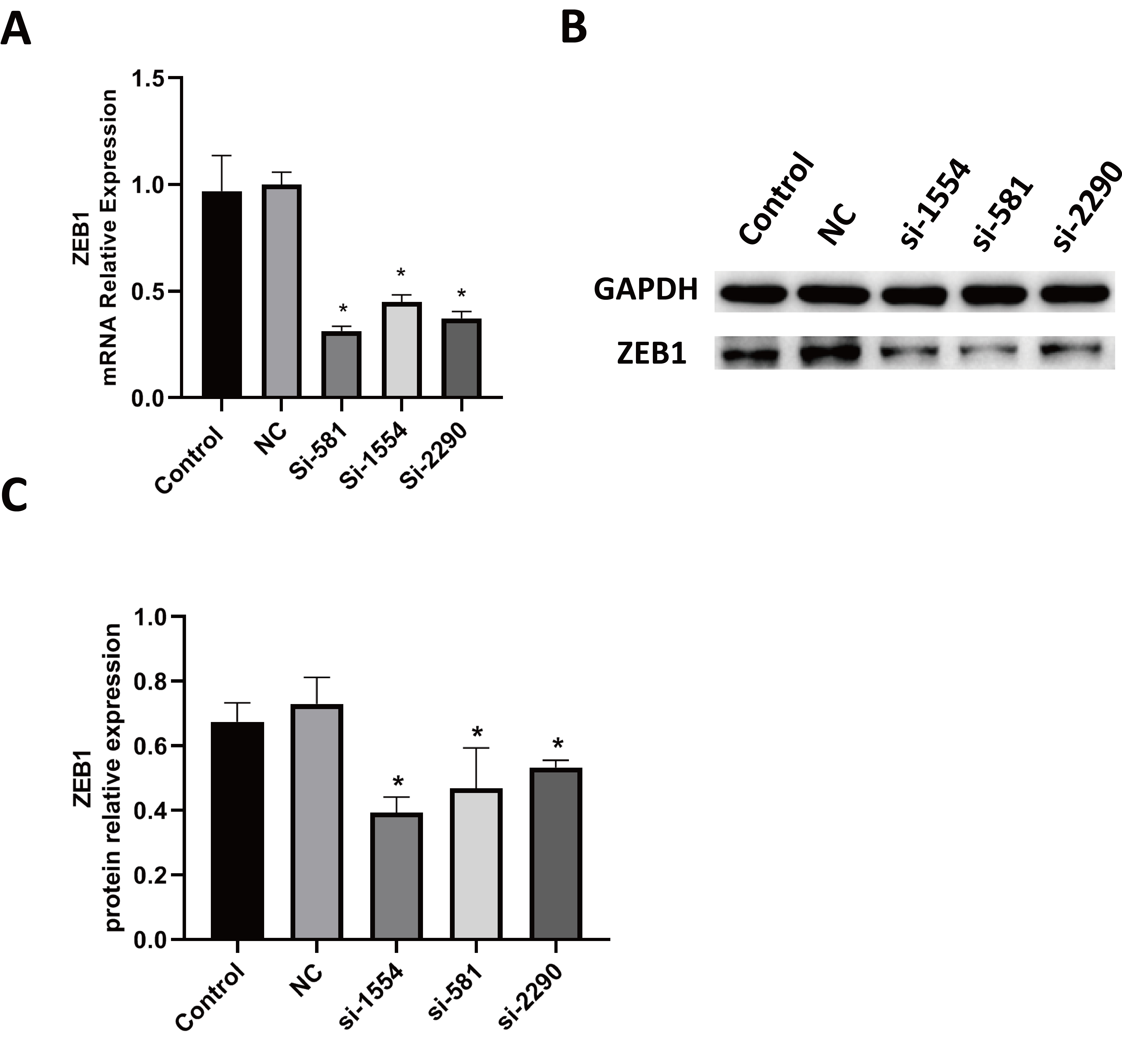

Supplement: Supplementary file 6 — Figure S6: Validation of ZEB1 knockdown efficiency in HK‐2 cells. (A) qRT‐PCR analysis of ZEB1 mRNA expression following transfection with three different ZEB1‐targeting siRNAs or a negative control (NC). (B) Western blot analysis of ZEB1 protein levels in the same conditions. (C) Densitometric quantification of ZEB1 protein expression normalised to GAPDH. Data are presented as mean ± SD (n = 3). *p < 0.05 versus control. [file JCMM-29-e70865-s003.jpg]

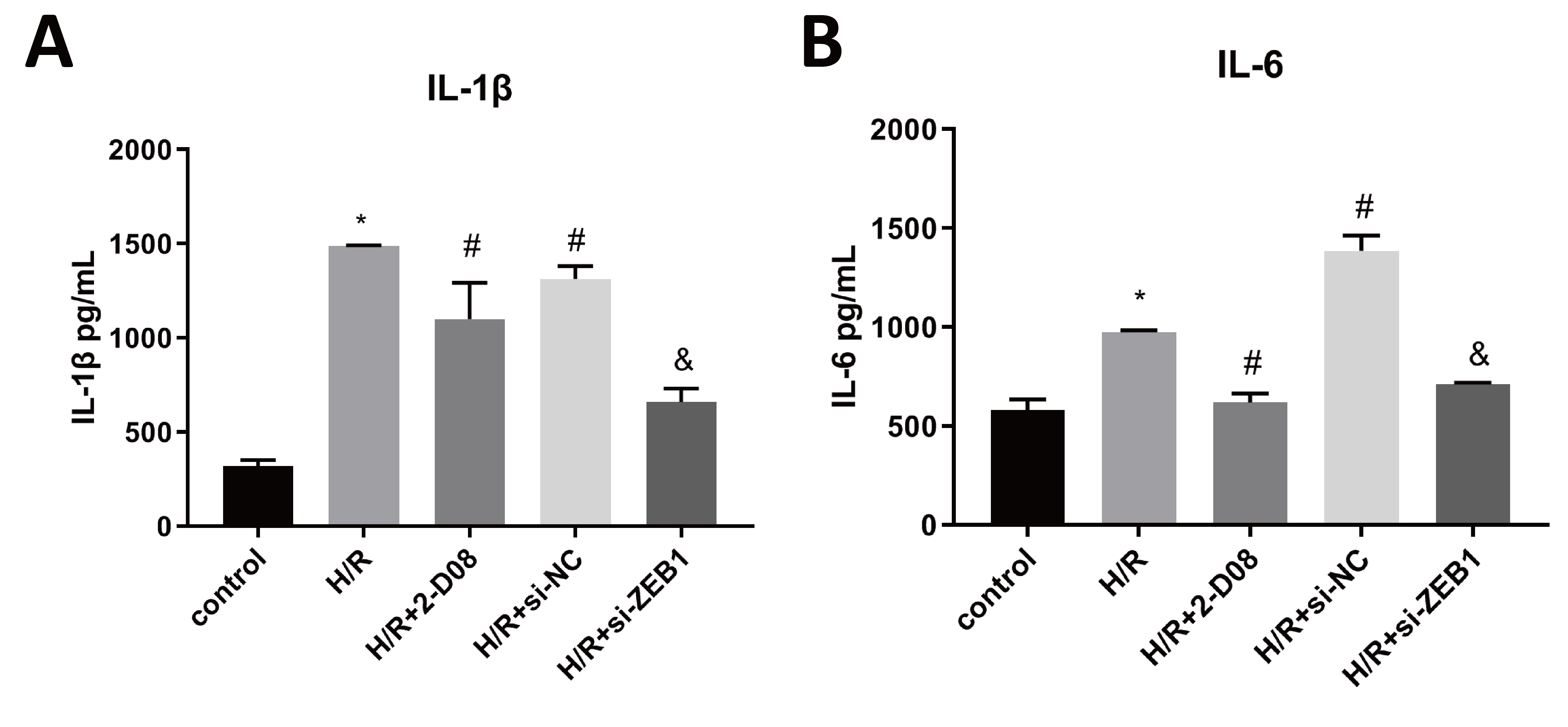

Supplement: Supplementary file 7 — Figure S7: Inflammatory cytokine levels in HK‐2 cells under H/R with ZEB1 knockdown or SUMOylation inhibition. (A) ELISA quantification of IL‐1β levels in culture supernatants from cells under five conditions: Control, H/R, H/R + 2‐D08, H/R + si‐NC and H/R + si‐ZEB1. (B) ELISA quantification of IL‐6 levels under the same treatment conditions. Data are presented as mean ± SD (n = 3). *p < 0.05 versus control; #p < 0.05 versus H/R; &p < 0.05 versus H/R + si‐NC. [file JCMM-29-e70865-s005.jpg]
