# Supplementary material for: SUMOylation of ZEB1 Modulates PANoptosis in Burn‐Induced Early Acute Kidney Injury
Source: J Cell Mol Med. 2025 Oct 22;29(20):e70865. doi: 10.1111/jcmm.70865 (PMC12544699; doi:10.1111/jcmm.70865)
Supplement: Supplementary file 8 — Table S1: Primers used in qPCR. [file JCMM-29-e70865-s007.docx]

Table S1 Primers used in qPCR

| Gene | Forward primer sequence (5’-3’) | Reverse primer sequence (5’-3’) |
| --- | --- | --- |
| β-actin | TGGCACCCAGCACAATGAA | CTAAGTCATAGTCCGCCTAGAAGCA |
| MLKL | GACTCCGCCTCAATTCTCCAT | CTGAATGGTGTAGCCGGTAT |
| Caspase-3 | ACAAATGGACCTGTTGACCTGA | TTGCTCCCATGTATGGTCTTTAC |
| GSDMD | GSDMD | GSDMD |
